# Wnt Signaling in Male Genital Lichen Sclerosus, Differentiated Penile Intraepithelial Neoplasia, and Penile Squamous Cell Carcinoma

**DOI:** 10.1016/j.xjidi.2025.100372

**Published:** 2025-04-11

**Authors:** Georgios Kravvas, Boyu Xie, Michael Millar, Alex Freeman, Aiman Haider, Hussain M. Alnajjar, Asif Muneer, Aamir Ahmed, Christopher Barry Bunker

**Affiliations:** 1 Department of Dermatology, University College London Hospitals NHS Foundation Trust, London, United Kingdom; 2 Cell and Developmental Biology, Division of Biosciences, University College London, London, United Kingdom; 3 The Queen’s Medical Research Institute, College of Medicine & Veterinary Medicine, University of Edinburgh, Edinburgh, United Kingdom; 4 Department of Histopathology, University College London Hospitals NHS Foundation Trust, London, United Kingdom; 5 Department of Urology, University College London Hospitals NHS Foundation Trust, London, United Kingdom

**Keywords:** Cancer biology, Squamous cell carcinoma, Wnt signaling

## Abstract

**Introduction:**

Male genital lichen sclerosus (MGLSc) is a chronic inflammatory disease causing scarring and significant morbidity, and predisposing individuals to differentiated penile intraepithelial neoplasia (dPeIN) and penile squamous cell carcinoma (PeSCC). Penile carcinogenesis follows two pathways: HPV-related and non-HPV-related. While HPV drives undifferentiated PeIN and warty/basaloid PeSCC, MGLSc is implicated in non-HPV-related dPeIN and "usual" PeSCC. Wnt signalling, pivotal in carcinogenesis, remains underexplored in MGLSc and PeIN.

**Methods:**

Tissue arrays from 114 archival samples of MGLSc, dPeIN, and PeSCC were analyzed using multi-label fluorescence staining and confocal microscopy for Wnt4, cyclin D1, c-MYC, and MMP7 expression.

**Results:**

Wnt signalling proteins were upregulated in PeSCC: cyclin D1 (2.3-fold), Wnt4 (2-fold), c-MYC (2.5-fold), and MMP7 (1.8-fold). Wnt4 expression increased in MGLSc (p=0.02), while dPeIN showed minimal changes. Altered co-localization of Wnt4/MMP7 (p=0.04) was observed in MGLSc and significant co-localization alterations of several protein pairs were also identified in PeSCC.

**Conclusion:**

Wnt signalling plays a role in progression from MGLSc to PeSCC through protein dysregulation. Overexpression and altered interactions in PeSCC highlight its potential as a diagnostic, prognostic, and therapeutic target.

## Introduction

Male genital lichen sclerosus (MGLSc) is a chronic, inflammatory, skin disorder characterized by progressive fibrosis and a significant impact on urological and sexual function. MGLSc also predisposes to penile intraepithelial neoplasia (PeIN) and penile squamous cell carcinoma (PeSCC) (Kravvas et al, 2018). Current evidence attributes the development of MGLSc to chronic exposure of the genital skin to occlusive urine contact (Kravvas et al., 2022a). Our recent research has shown that transcriptionally active human papillomavirus (HPV) likely does not contribute to the pathogenesis of MGLSc or differentiated PeIN (dPeIN), although it remains a key factor in the development of undifferentiated PeIN (Kravvas et al, 2024). Emerging evidence supports distinct etiological pathways for HPV-related and MGLSc-related malignancies: whereas undifferentiated PeIN and warty or basaloid PeSCC are linked to HPV infection, dPeIN and “usual” or verrucous PeSCC are more strongly associated with MGLSc (Bunker, 2019; Bunker and Porter, 2016; Kravvas et al., 2022b).

The Wnt pathway is a key signaling cascade in most eukaryotes throughout their lifespan (Buechling and Boutros, 2011; Croce and McClay, 2008). Wnt signaling plays a central role in many developmental processes, such as cell fate specification during early embryonic development, body axis patterning, and tissue homeostasis (Clevers, 2006; Gu et al, 2010; Nusse, 2008). Dysregulation in Wnt signaling has also been associated with inflammatory processes and a variety of diseases, including cancers (Arya et al, 2015; Bienz and Clevers, 2000; Clevers, 2004; Polakis, 2012).

Target genes of Wnt/ß-catenin transcription, CD1, c-MYC, and matrix metalloproteinase 7 (MMP7), are considered proto-oncogenes (Ashley et al, 2021). Owing to the activation of transcription of these and other genes, Wnt signaling is considered a critical step in carcinogenesis, including in carcinomas of the ovary, colon, and prostate and in melanoma and squamous cell carcinoma (Arya et al, 2015; Brabletz et al, 1999; Giuliano et al, 2016; Hofmann et al, 2006; Niehrs and Acebron, 2012; Wang et al, 2010; Zeng et al, 2006). Thus, overexpression of proteins encoded by Wnt/ß-catenin–target genes has been used as a surrogate to determine the role of Wnt signaling in human carcinomas (Lin et al, 2000).

It has also been postulated that Wnt/ß-catenin signaling may be a key component of PeCa carcinogenesis (Arya et al, 2015). Arya et al (2015) showed that WNT4, MMP7, CD1, and c-MYC increased significantly in PeCa compared with those in normal penile tissue. This was further strengthened by their observation that there was additionally a significant difference in the colocalization of c-MYC and CD1 in PeCa carcinoma samples compared with those in controls (Arya et al, 2015). We postulate that investigating the role of Wnt signaling in MGLSc and its potential influence on the progression to penile carcinogenesis could provide valuable insights into the pathogenesis of the disease and inform future prognostic and management strategies.

## Results

### Patient characteristics

A total of 114 patients were recruited. This included 48 men with MGLSc, 21 with dPeIN, and 23 with PeSCC. All cases of PeSCC included in this study were of the ‘usual’ subtype. However, for optimal intelligibility, this qualifier has been largely omitted from the rest of this report.

### Protein expression in MGLSc, dPeIN, and PeSCC

**Figure 1:**
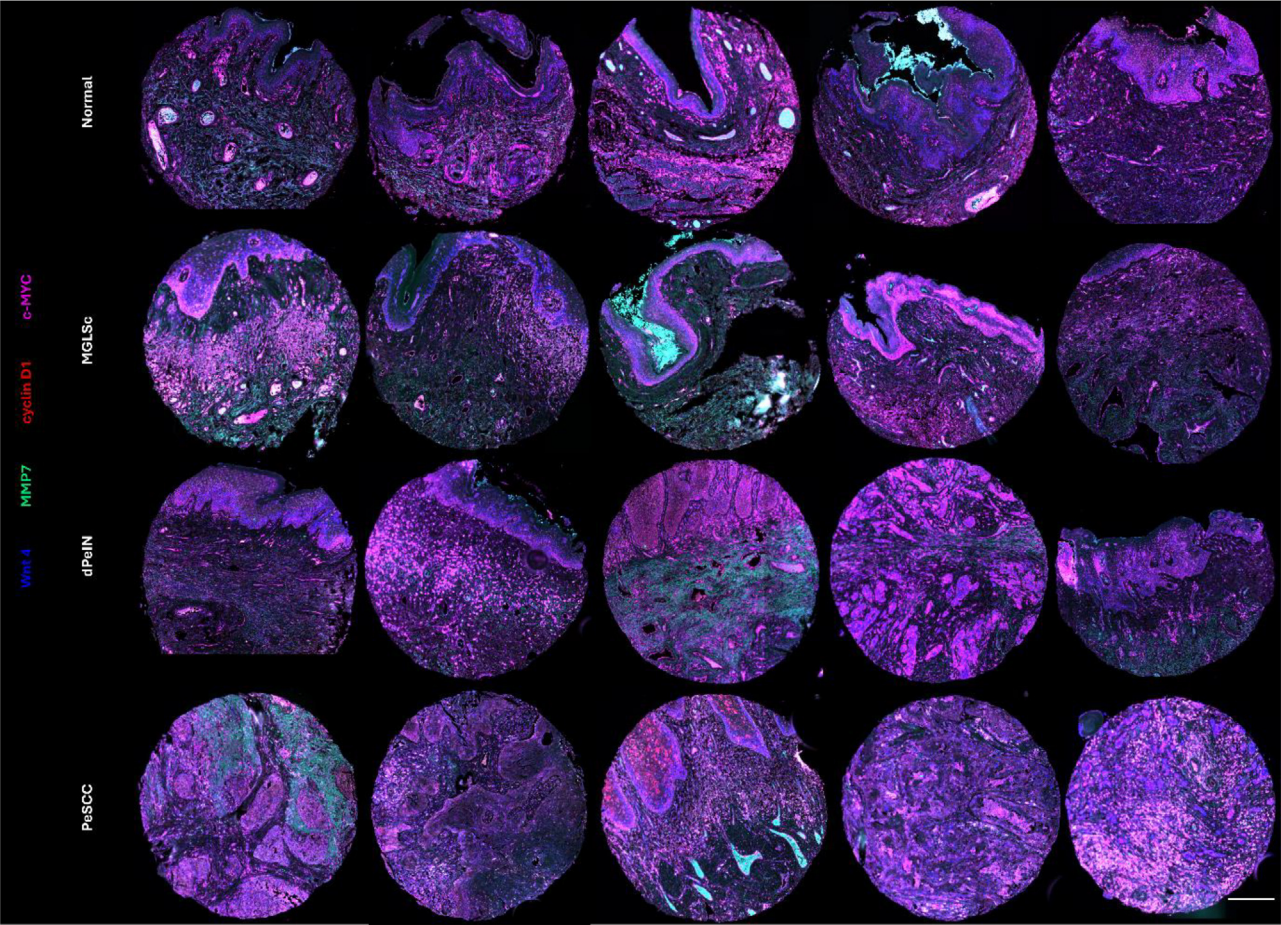
**Multilabeled immunofluorescence TA cores of normal penile skin, MGLSc, dPeIN, and PeSCC.** Composite views of the types of tissues used in this study, probed with Wnt4 (blue, Opal 480 labeled), MMP7 (green, Opal 520 labeled), cyclin D1 (red, Opal 570 labeled), and c-MYC (pink, Opal 650 labeled). Each row represents 5 tissue cores for normal, MGLSc, dPeIN, and PeSCC (from top to bottom). All the settings are kept the same for a comparative analysis. Tissue core diameter: 1 mm. Bar = 600 μm. dPeIN, differentiated penile intraepithelial neoplasia; MGLSc, male genital lichen sclerosus; MMP7, matrix metalloproteinase 7; PeSCC, penile squamous cell carcinoma; TA, tissue array.

**Figure 2:**
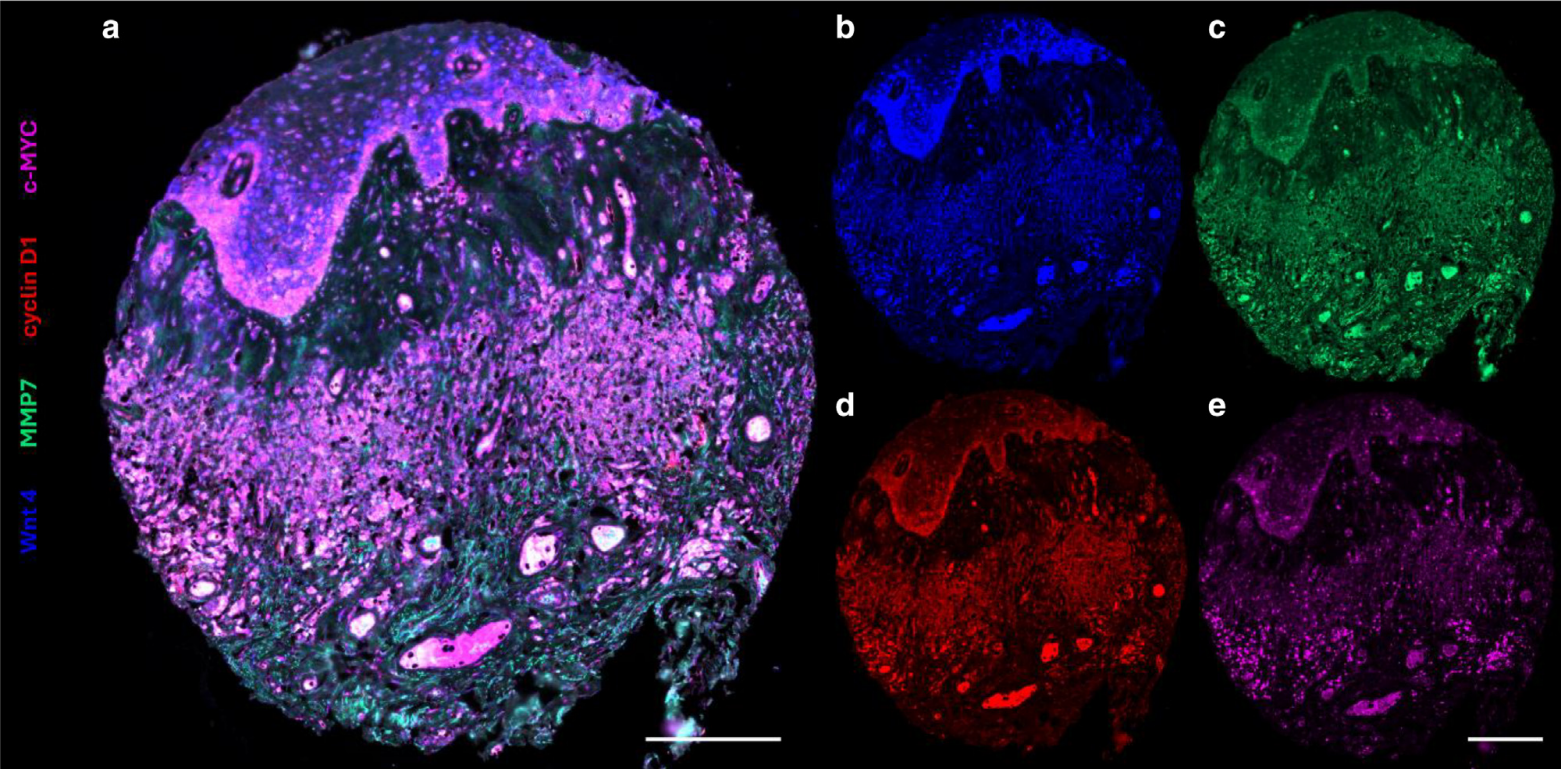
**A single multilabeled immunofluorescence TA core of MGLSc.** An example of a tissue core stained for cyclin D1, Wnt4, c-MYC, and MMP7. (**a**) Composite channels. (**b–e**) Single channel views: (**b**) Wnt4 (blue, Opal 480 labeled), (**c**) MMP7 (green, Opal 520 labeled), (**d**) cyclin D1 (red, Opal 570 labeled), (**e**) c-MYC (pink, Opal 650 labeled). The images were taken with the Zeiss AxioScan Z1 slide scanner at ×20 magnification. All the settings were kept the same for a comparative analysis. Tissue core diameter: 1 mm. The composite image **a** is an amalgamation of the 4 individual channels **b–e** and may not readily be visualized as conventional mixture of the 4 colors; each channel is objectively quantified using an algorithm as described in Materials and Methods. Both bars = 600 μm. MGLSc, male genital lichen sclerosus; MMP7, matrix metalloproteinase 7; TA, tissue array.

Representative composite images of tissue cores demonstrating the expression of Wnt4, MMP7, cyclin D1, and c-MYC in normal tissue, MGLSc, dPeIN, and PeSCC are shown in Figure 1. These images integrate 4 fluorescent channels, with individual channel images for each of the 4 fluorophores provided separately in Figure 2.

**Figure 3:**
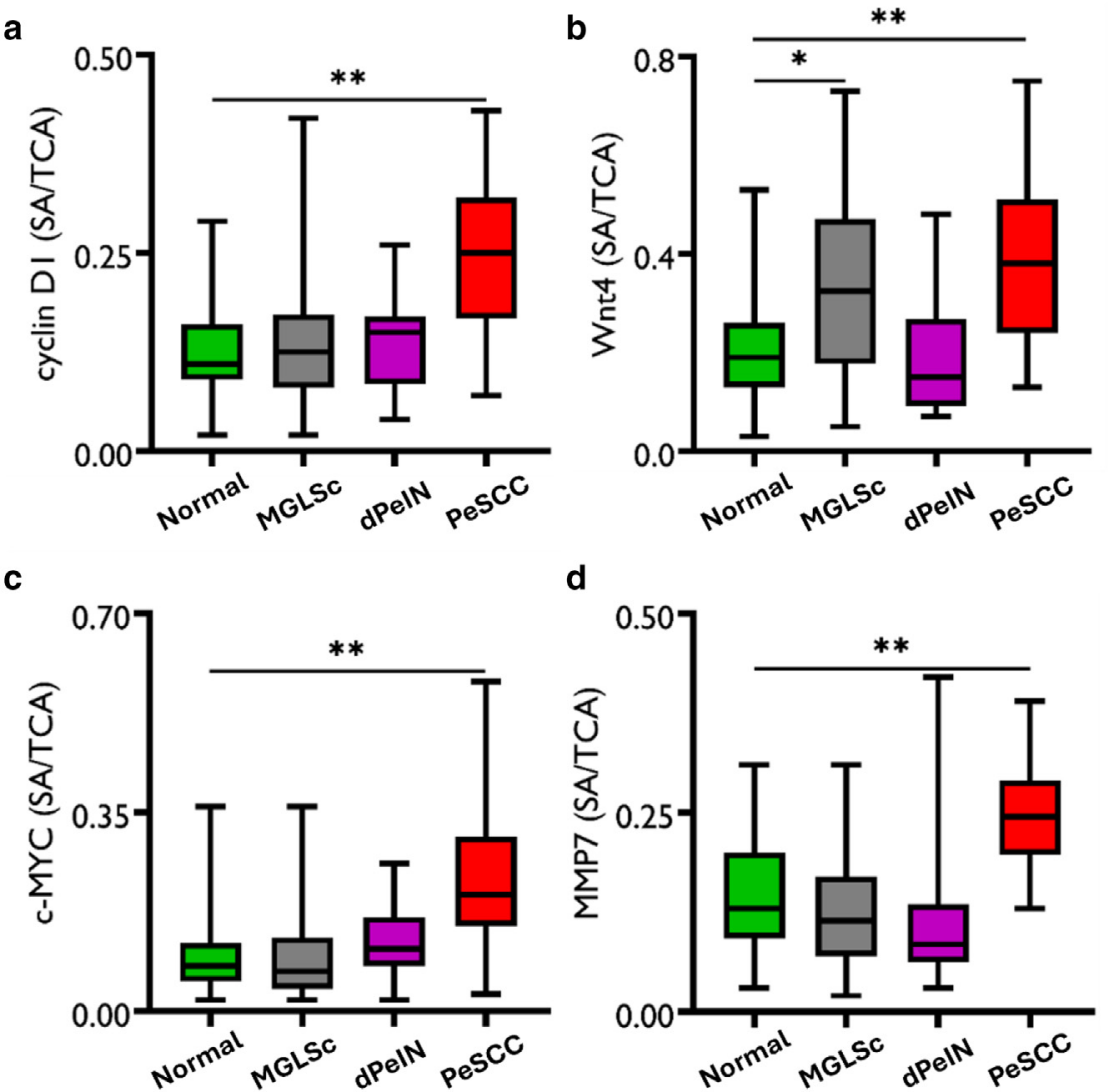
**Boxplots for the quantitative expression of cyclin D1, Wnt4, c-MYC, and MMP7 in normal penile skin compared with those in MGLSc, dPeIN, and PeSCC.** (**a**) Cyclin D1, **(b)** Wnt4, **(c)** c-MYC, and **(d)** MMP7. Quantitative expression of the 4 Wnt-related proteins ([**a**] cyclin D1, [**b**] Wnt4, [**c**] c-MYC, and [**d**] MMP7) in normal penile skin compared with those in MGLSc, dPeIN, and PeSCC. Green rectangles represent normal penile skin (N = 36, n = 36), gray rectangles represent MGLSc (N = 46, n = 46), purple rectangles represent dPeIN (N = 20, n = 20), and red rectangles represent PeSCC (N = 18, n = 18). The boxplots show the median, minimum, maximum, and 25 and 75% interquartile ranges for each group. The results for intensity are presented as SA divided by the TCA (SA/TCA). The SA was divided by the TCA to standardize the data for the variable amounts of tissue that were present in each core. (N = number of patients; n = number of tissue cores; ∗*P* ≤ .05 and ∗∗*P* ≤ .01). dPeIN, differentiated penile intraepithelial neoplasia; MGLSc, male genital lichen sclerosus; MMP7, matrix metalloproteinase 7; PeSCC, penile squamous cell carcinoma; SA, signal area; TCA, total core area.

When compared with normal tissues, the expression of cyclin D1 in PeSCC was increased by 2.3 fold, the expression of Wnt4 was increased by 1.7 fold in MGLSc and by 2 fold in PeSCC, the expression of c-MYC was increased by 2.5 fold in PeSCC, and the expression of MMP7 was increased by 1.8 fold in PeSCC (Table 1 and Figure 3).

**Table 1:** The Expression of Cyclin D1, Wnt4, c-MYC, and MMP7 in Normal Penile Skin Compared with that in MGLSc, PeIN, and PeSCC

Condition	Cyclin D1	Wnt4	c-MYC	MMP7
Normal	0.11 (0.09, 0.16)	0.19 (0.13, 0.26)	0.08 (0.05, 0.12)	0.13 (0.090, 0.200)
MGLSc	0.12 (0.08, 0.17)	0.32 (0.18, 0.46)	0.07 (0.04, 0.13)	0.11 (0.07, 0.170)
dPeIN	0.15 (0.09, 0.17)	0.15 (0.09, 0.26)	0.11 (0.08, 0.16)	0.08 (0.06, 0.13)
PeSCC	0.25 (0.17, 0.31)	0.38 (0.25, 0.48)	0.20 (0.15, 0.29)	0.24 (0.20, 0.29)
Normal versus MGLSc	*P* = 1	***P* = .02**	*P* = 1	*P* = 1
Normal versus dPeIN	*P* = 1	*P* = 1	*P* = .9	*P* = .3
Normal versus PeSCC	***P* = .003**	***P* = .008**	***P* = .003**	***P* = .003**

Abbreviations: dPeIN, differentiated penile intraepithelial neoplasia; MGLSc, male genital lichen sclerosus; MMP7, matrix metalloproteinase 7; PeSCC, penile squamous cell carcinoma.

The expression values of each of the 4 Wnt-related proteins (cyclin D1, Wnt4, c-MYC, and MMP7) in normal penile skin (N = 36, n = 36), MGLSc (N = 46, n = 46), dPeIN (N = 20, n = 20), and PeSCC (N = 18, n = 18) are presented as median values and (25%, 75% interquartile). In addition, Holm–Bonferroni–adjusted *P*-values are given for each condition compared with normal. *P*-values in bold font signify statistically significant results (N = number of patients; n = number of tissue cores).

### Protein colocalization in MGLSc, dPeIN, and PeSCC

**Figure 4:**
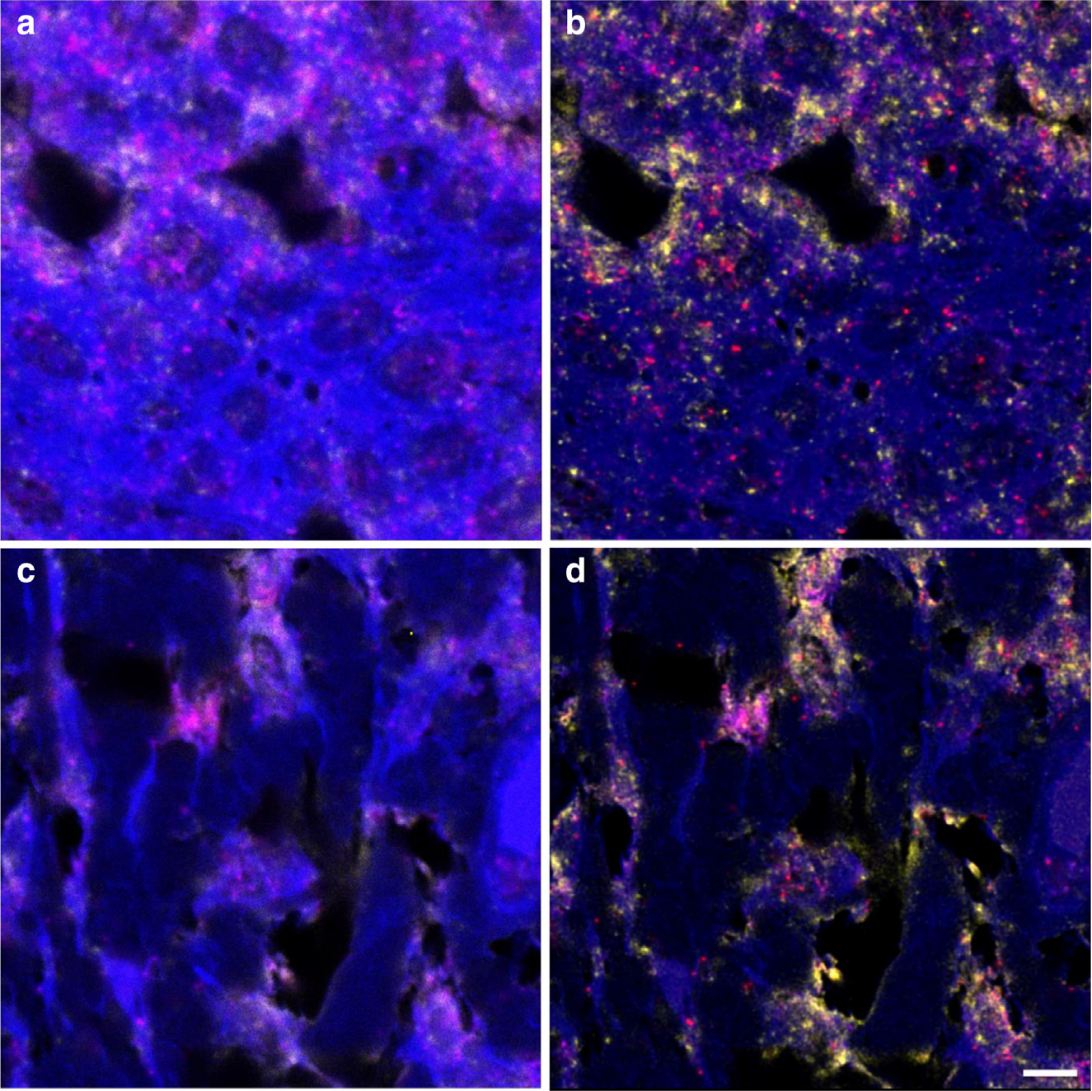
**Multilabeled confocal images of a tissue core.** (**a**) Epidermis. (**b**) Deconvolved view of the epidermis. (**c**) Dermis. (**d**) Deconvolved view of the dermis. Presented are Wnt4 (blue, Opal 480 labeled), MMP7 (yellow, Opal 520 labeled), cyclin D1 (red, Opal 570 labeled), and c-MYC (pink, Opal 650 labeled). The images were taken with the Leica SP8 confocal microscope at ×63 magnification and were subsequently deconvolved using Huygens Professional software. Bar = 20 μm. MMP7, matrix metalloproteinase 7.

The small surface area of the high-power images taken by the confocal microscope, coupled with the potential to choose which structures of a core were being imaged, enabled a segregated analysis of protein colocalization between the epidermis and dermis (Figure 4).

**Figure 5:**
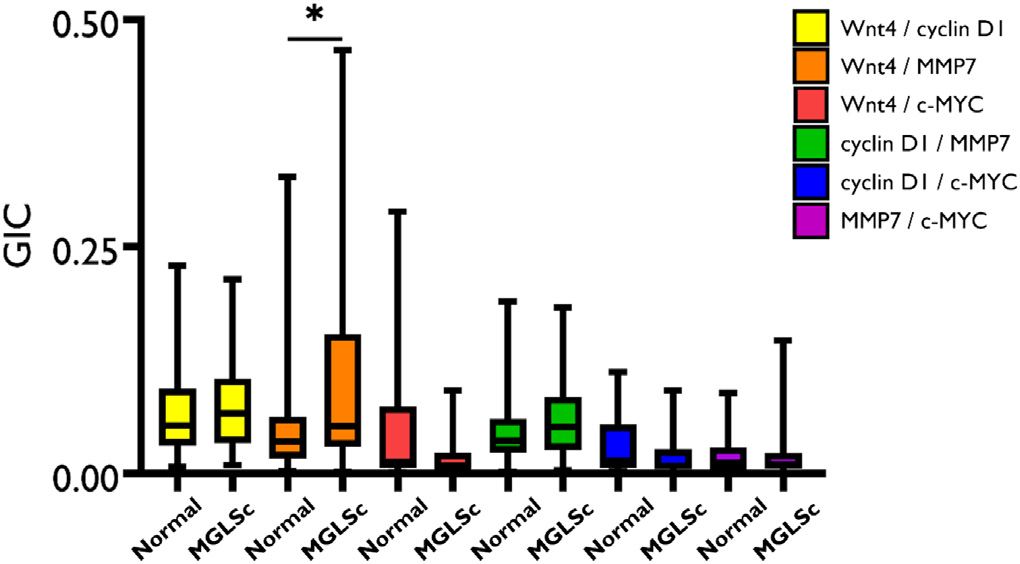
**Boxplots for the colocalization of the 6 protein pairs (Wnt4/cyclin D1, Wnt4/MMP7, Wnt4/c-MYC, cyclin D1/MMP7, cyclin D1/c-MYC, and MMP7/c-MYC) in the epidermis, in normal tissues, and in MGLSc.** Shown is a protein colocalization analysis of the 6 protein pairs in normal tissues (N = 32, n = 32) and MGLSc (N = 29, n = 29). The boxplots show the median, minimum, maximum, and 25 and 75% interquartile ranges for each group. Yellow = Wnt/cyclin D1, orange = Wnt4/MMP7, red = Wnt 4/c-MYC, green = cyclin D1/MMP7, blue = cyclin D1/MYC, and purple = MMP7/c-MYC. Colocalization values are expressed as GICs (N = number of patients; n = number of tissue cores; ∗*P* ≤ .05). GIC, Global Intersection Coefficient; MGLSc, male genital lichen sclerosus; MMP7, matrix metalloproteinase 7.

**Figure 6:**
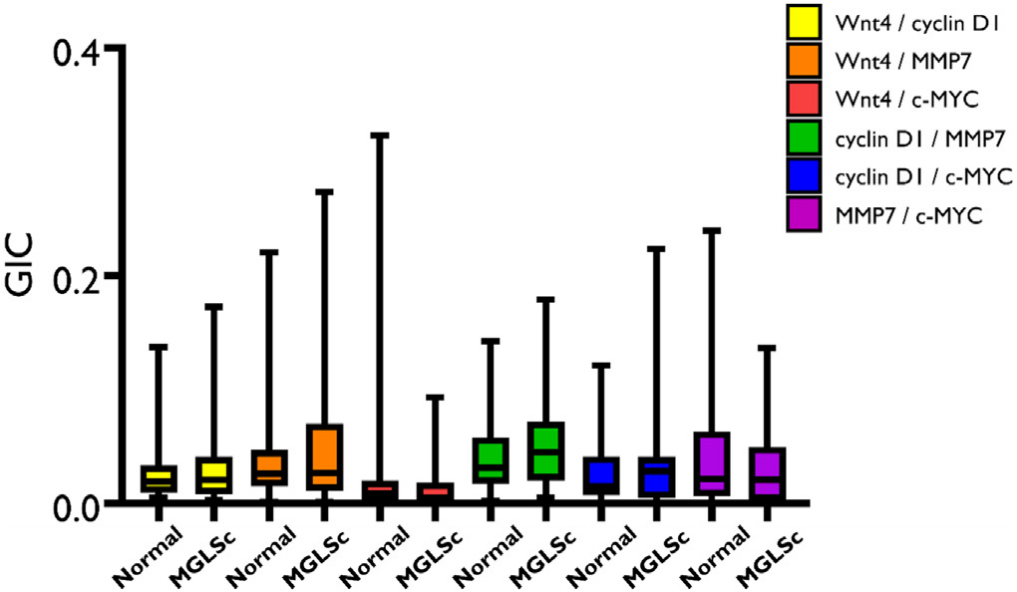
**Boxplots for the colocalization of the 6 protein pairs (Wnt4/cyclin D1, Wnt4/MMP7, Wnt4/c-MYC, cyclin D1/MMP7, cyclin D1/c-MYC, and MMP7/c-MYC) in the dermis, in normal tissues, and in MGLSc.** Shown is a colocalization of the 6 protein pairs in normal tissues (N = 32, n = 32) and MGLSc (N = 32, n = 32). The boxplots show the median, minimum, maximum, and 25 and 75% interquartile ranges for each group. Yellow = Wnt/cyclin D1, orange = Wnt4/MMP7, red = Wnt 4/c-MYC, green = cyclin D1/MMP7, blue = cyclin D1/MYC, and purple = MMP7/c-MYC. Colocalization values are expressed as GICs (N = number of patients; n = number of tissue cores). GIC, Global Intersection Coefficient; MGLSc, male genital lichen sclerosus; MMP7, matrix metalloproteinase 7.

**Figure 7:**
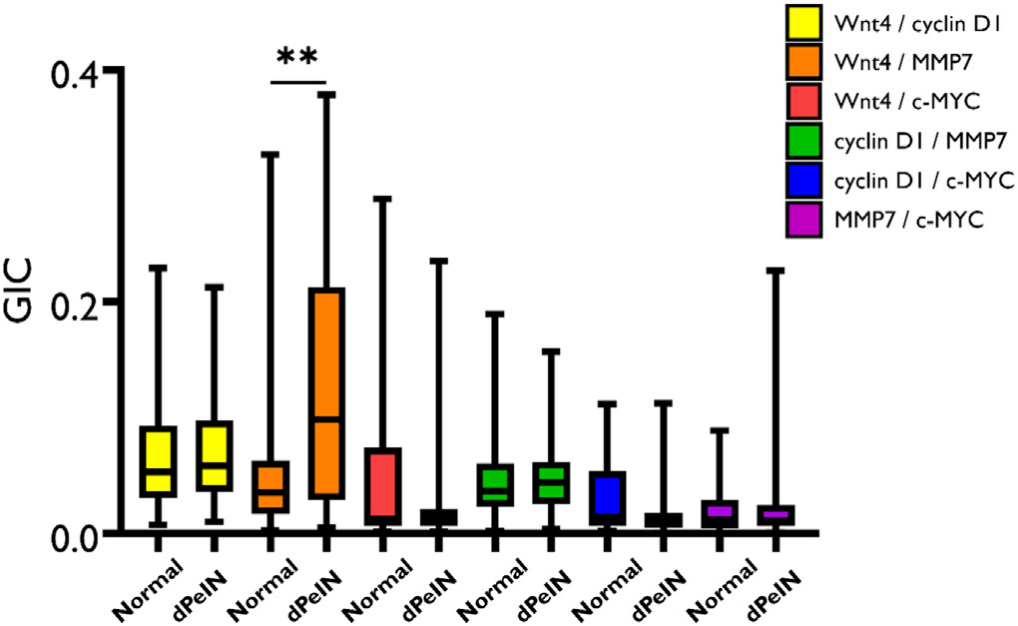
**Boxplots for the colocalization of the 6 protein pairs (Wnt4/cyclin D1, Wnt4/MMP7, Wnt4/c-MYC, cyclin D1/MMP7, cyclin D1/c-MYC, and MMP7/c-MYC) in the epidermis, in normal tissues, and in dPeIN.** Shown is a colocalization of the 6 protein pairs in normal tissues (N = 32, n = 32) and dPeIN (N = 16, n = 16). The boxplots show the median, minimum, maximum, and 25 and 75% interquartile ranges for each group. Yellow = Wnt/cyclin D1, orange = Wnt4/MMP7, red = Wnt 4/c-MYC, green = cyclin D1/MMP7, blue = cyclin D1/MYC, and purple = MMP7/c-MYC. Colocalization values are expressed as GICs (N = number of patients; n = number of tissue cores; ∗∗*P* ≤ .01). dPeIN, differentiated penile intraepithelial neoplasia; GIC, Global Intersection Coefficient; MMP7, matrix metalloproteinase 7.

**Figure 8:**
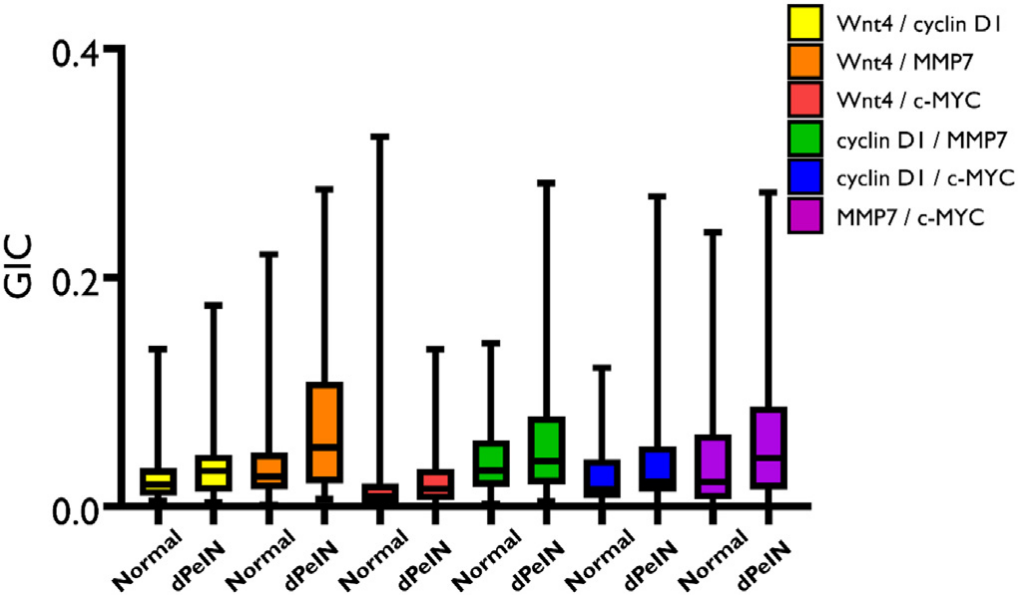
**Boxplots for the colocalization of the 6 protein pairs (Wnt4/cyclin D1, Wnt4/MMP7, Wnt4/c-MYC, cyclin D1/MMP7, cyclin D1/c-MYC, and MMP7/c-MYC) in the dermis, in normal tissues, and in dPeIN.** Shown is a colocalization of the 6 protein pairs in normal tissues (N = 32, n = 32) and dPeIN (N = 18, n = 18). The boxplots show the median, minimum, maximum, and 25 and 75% interquartile ranges for each group. Yellow = Wnt/cyclin D1, orange = Wnt4/MMP7, red = Wnt 4/c-MYC, green = cyclin D1/MMP7, blue = cyclin D1/MYC, and purple = MMP7/c-MYC. Colocalization values are expressed as GICs (N = number of patients; n = number of tissue cores). dPeIN, differentiated penile intraepithelial neoplasia; GIC, Global Intersection Coefficient; MMP7, matrix metalloproteinase 7.

When compared with normal skin, MGLSc and dPeIN were found to possess very few alterations in protein colocalization. Specifically, in MGLSc, the colocalization of one protein pair (Wnt4/MMP7) was altered in the epidermis, and the colocalization of none was in the dermis (details are provided in the Materials and Methods). In dPeIN, significant alterations in protein colocalization were found in one protein pair in the epidermis (Wnt4/MMP7), and none was found in the dermis (Figure 5, Figure 6, Figure 7, Figure 8).

**Figure 9:**
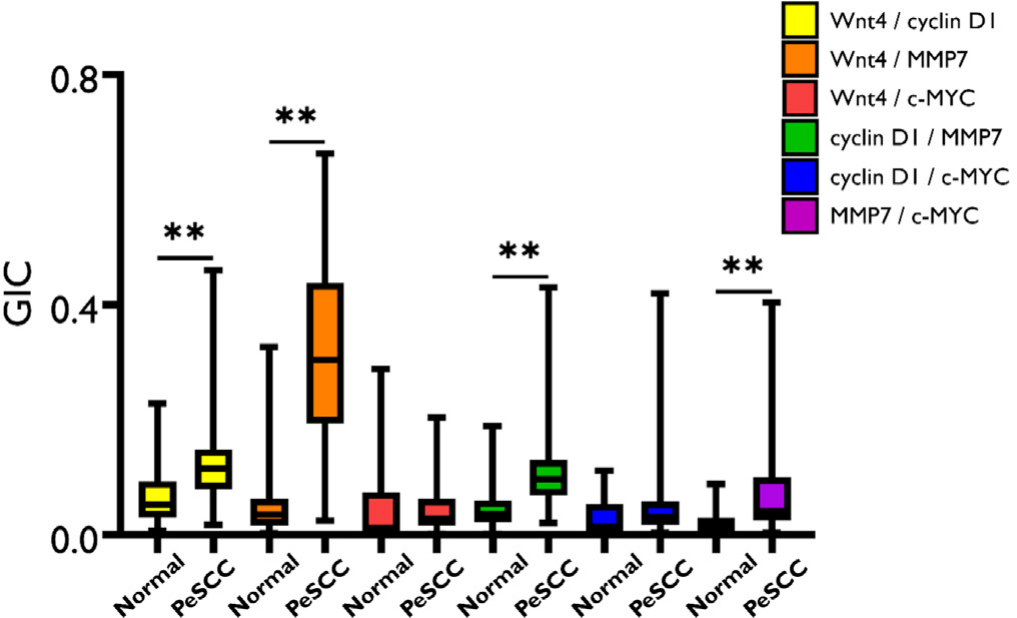
**Boxplots for the colocalization of the 6 protein pairs (Wnt4/cyclin D1, Wnt4/MMP7, Wnt4/c-MYC, cyclin D1/MMP7, cyclin D1/c-MYC, and MMP7/c-MYC) in the epidermis, in normal tissues, and in PeSCC.** Presented is a colocalization of the 6 protein pairs in normal tissues (N = 32, n = 32) and PeSCC (N = 21, n = 21). The boxplots show the median, minimum, maximum, and 25 and 75% interquartile ranges for each group. Yellow = Wnt/cyclin D1, orange = Wnt4/MMP7, red = Wnt 4/c-MYC, green = cyclin D1/MMP7, blue = cyclin D1/MYC, and purple = MMP7/c-MYC. Colocalization values are expressed as GICs (N = number of patients; n = number of tissue cores. ∗∗*P* ≤ .01). GIC, Global Intersection Coefficient; MMP7, matrix metalloproteinase 7; PeSCC, penile squamous cell carcinoma.

**Figure 10:**
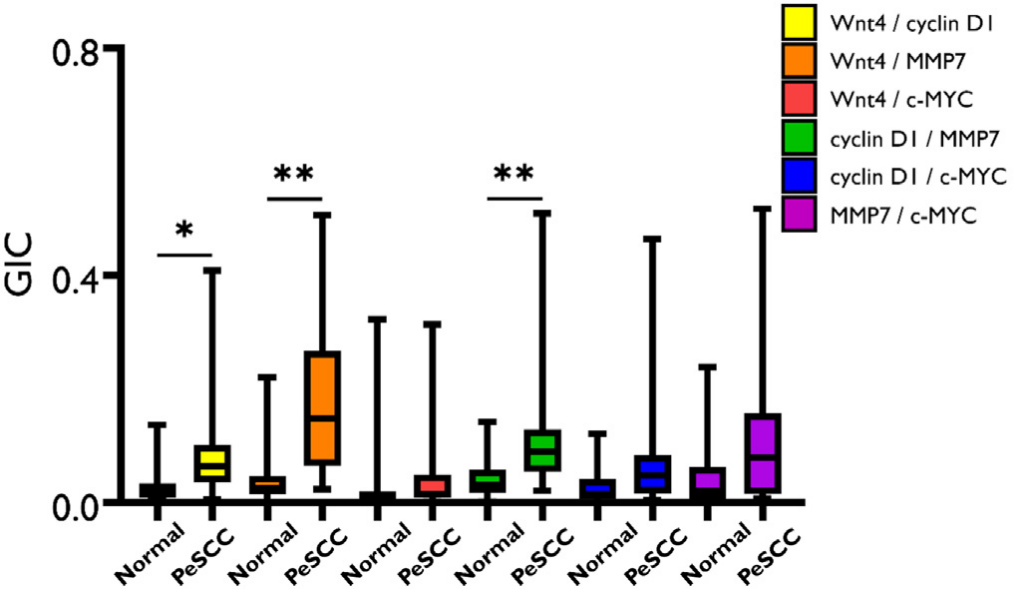
**Boxplots for the colocalization of the 6 protein pairs (Wnt4/cyclin D1, Wnt4/MMP7, Wnt4/c-MYC, cyclin D1/MMP7, cyclin D1/c-MYC, and MMP7/c-MYC) in the dermis, in normal tissues, and in PeSCC.** Shown is a colocalization of the 6 protein pairs in normal tissues (N = 32, n = 32) and PeSCC (N = 17, n = 17). The boxplots show the median, minimum, maximum, and 25 and 75% interquartile ranges for each group. Yellow = Wnt/cyclin D1, orange = Wnt4/MMP7, red = Wnt 4/c-MYC, green = cyclin D1/MMP7, blue = cyclin D1/MYC, and purple = MMP7/c-MYC. Colocalization values are expressed as GICs (N = number of patients; n = number of tissue cores. ∗*P* ≤ .05 and ∗∗*P* ≤ .01). GIC, Global Intersection Coefficient; MMP7, matrix metalloproteinase 7; PeSCC, penile squamous cell carcinoma.

However, in PeSCC, protein colocalization was found to be significantly altered across multiple protein pairs. To elaborate, alterations were identified in the colocalization of 4 protein pairs in the epidermis (Wnt4/cyclin-D1, Wn4/MMP7, cyclin D1/MMP7, and MMP7/c-MYC) and in 3 protein pairs in the dermis (Wnt4/cyclin-D1, Wn4/MMP7, and cyclin D1/MMP7) (Figures 9 and 10).

A summary of all the colocalization results in the epidermis and dermis is presented in Tables 2 and 3.

**Table 2:** The Colocalization of the 6 Protein Pairs—Wnt4/Cyclin D1, Wnt4/MMP7, Wnt4/c-MYC, Cyclin D1/MMP7, Cyclin D1/c-MYC, and MMP7/c-MYC—in the Epidermis

Condition	Wnt4/Cyclin D1	Wnt4/MMP7	Wnt4/c-MYC	Cyclin D1/MMP7	Cyclin D1/c-MYC	MMP7/c-MYC
Normal	0.05 (0.03, 0.09)	0.03 (0.01, 0.06)	0.01 (0.01, 0.07)	0.03 (0.2, 0.6)	0.01 (0.01, 0.05)	0.01 (0.01, 0.02)
MGLSc	0.06 (0.03, 0.10)	0.05 (0.03, 0.15)	0.01 (0.01, 0.02)	0.05 (0.02, 0.08)	0.01 (0.01, 0.02)	0.01 (0.01, 0.02)
dPeIN	0.05 (0.03, 0.09)	0.09 (0.02, 0.21)	0.01 (0.01, 0.02)	0.04 (0.02, 0.06)	0.01 (0.01, 0.01)	0.01 (0.01, 0.02)
PeSCC	0.11 (0.07, 0.14)	0.30 (0.19, 0.43)	0.02 (0.01, 0.06)	0.09 (0.06, 0.12)	0.03 (0.01, 0.05)	0.04 (0.02, 0.09)
Normal versus MGLSc	*P* = 1	***P* = .04**	*P* = .1	*P*' = 1	*P* = .3	*P* = 1
Normal versus dPeIN	*P* = 1	***P* = .01**	*P* = 1	*P* = 1	*P* = .3	*P* = 1
Normal versus PeSCC	***P* = .006**	***P* = .006**	*P* = 1	***P* = .006**	*P* = .1	***P* = .006**

Abbreviations: adj., adjacent; dPeIN, differentiated penile intraepithelial neoplasia; GIC, Global Intersection Coefficient; MGLSc, male genital lichen sclerosus; MMP7, matrix metalloproteinase 7; PeSCC, penile squamous cell carcinoma.

The GIC values of each of the 6 protein pairs for normal skin (N = 32, n = 32), MGLSc (N = 29, n = 29), dPeIN (N = 16, n = 16), and PeSCC (N = 21, n = 21) are presented as median values and (25%, 75% interquartile). In addition, Holm–Bonferroni–adjusted *P*-values are given for the differences in colocalization. *P*-values in bold font signify statistically significant results (N = number of patients; n = number of tissue cores).

**Table 3:** The Colocalization of the 6 Protein Pairs—Wnt4/Cyclin D1, Wnt4/MMP7, Wnt4/c-MYC, Cyclin D1/MMP7, Cyclin D1/c-MYC, and MMP7/c-MYC—in the Dermis

Condition	Wnt4/Cyclin D1	Wnt4/MMP7	Wnt4/c-MYC	Cyclin D1/MMP7	Cyclin D1/c-MYC	MMP7/c-MYC
Normal	0.02 (0.01, 0.03)	0.03 (0.01, 0.05)	0.01 (0.00, 0.02)	0.03 (0.02, 0.06)	0.01 (0.01, 0.04)	0.02 (0.01, 0.06)
MGLSc	0.02 (0.01, 0.04)	0.03 (0.01, 0.07)	0.00 (0.00, 0.02)	0.04 (0.02, 0.07)	0.03 (0.00, 0.04)	0.02 (0.00, 0.05)
dPeIN	0.03 (0.01, 0.04)	0.05 (0.02, 0.10)	0.01 (0.01, 0.03)	0.04 (0.02, 0.07)	0.02 (0.01, 0.05)	0.04 (0.02, 0.09)
PeSCC	0.06 (0.04, 0.10)	0.15 (0.07, 0.25)	0.02 (0.01, 0.05)	0.10 (0.06, 0.13)	0.05 (0.02, 0.08)	0.08 (0.02, 0.16)
Normal versus MGLSc	*P* = 1	*P* = 1	*P* = 1	*P* = 1	*P* = 1	*P* = 1
Normal versus dPeIN	*P* = 1	***P* = .09**	*P* = 1	*P* = 1	*P* = 1	*P* = .5
Normal versus PeSCC	***P* = .01**	***P* = .006**	*P* = 1	***P* = .006**	*P* = .1	*P* = .3

Abbreviations: adj., adjacent; dPeIN, differentiated penile intraepithelial neoplasia; GIC, Global Intersection Coefficient; MGLSc, male genital lichen sclerosus; MMP7, matrix metalloproteinase 7; PeSCC, penile squamous cell carcinoma.

The GIC values of each of the 6 protein pairs for normal (N = 32, n = 32), PeSCC adj. (N = 8, n = 8), MGLSc (N = 32, n = 32), dPeIN (N = 18, n = 18), and PeSCC (N = 17, n = 17) are presented as median values and (25%, 75% interquartile). In addition, Holm–Bonferroni adjusted *P*-values are given for the differences in colocalization. *P*-values in bold font signify statistically significant results (N = number of patients; n = number of tissue cores).

### Summary

Protein expression in MGLSc and dPeIN was not significantly altered compared with that in normal skin; however, there was an increase in Wnt4 expression in MGLSc. The expression of all 4 Wnt-related proteins was found to be increased 1.8–2.5 folds in PeSCC. In MGLSc and dPeIN, significant alterations in the colocalization of only 1 protein pair were seen in the epidermis and of none in the dermis. In PeSCC, significant alterations in protein colocalization were found in 4 protein pairs in the epidermis and in 3 protein pairs in the dermis.

## Discussion

Wnt signaling has been implicated in inflammatory processes and in PeSCC but has not yet been studied in MGLSc or PeIN (Kim et al, 2022). MGLSc is an inflammatory and scarring condition, and it is therefore rational to suppose that dysregulation of Wnt signaling may occur.

In this study, both increased expression of Wnt4 and altered epidermal colocalization of Wnt4/MMP7 were measured in MGLSc. The findings support the idea that Wnt-related dysregulation could contribute to or be a result of inflammation and scarring and could serve as useful biomarkers for MGLSc and PeSCC. However, these data must be interpreted with care as discussed below.

Wnt signaling is activated by binding of Wnt ligand proteins to their receptors regulated by the prevailing cell membrane potential (Ashmore et al, 2019). Wnt signal activation initiates a cascade of intracellular events, mainly release of free calcium and stabilization and nuclear translocation of a transcription factor coactivator protein, β-catenin (Thrasivoulou et al, 2013). Combining with TCF/LEF proteins, β-catenin promotes gene transcription of numerous genes, including proto-oncogenes, involved in proliferation, inflammation, and other cellular processes (Song et al, 2024). A limitation of our study is that we only investigated the expression of protein products of a very small number of these genes (namely, Wnt4, MMP7, cyclin D1, and c-MYC). None of the targets of Wnt/β-catenin transcription showed a change in expression even though the expression of the ligand Wnt4 was increased in MGLSc samples. Although an absence of increase in some proteins transcribed by the Wnt target genes in MGLSc and dPeIN suggests that there may not be a role for Wnt signaling in the progression of MGLSc to penile cancer, this interpretation is necessarily limited by the small number of targets of Wnt/β-catenin transcriptions tested in this study. It is still demonstrably the case that the pathway to PeSCC from MGLSc through dPeIN can be partially described by dysregulation of Wnt signaling. Our results add weight to the notion that proteins of the Wnt signaling pathway could be useful biomarkers for the diagnosis and prognostic stratification of disease as well as potential targets for therapy (Arya et al, 2015). This idea is strengthened because colocalization of Wnt-related proteins show a distinct pattern of significant alteration in the different disease conditions investigated in this report.

Dysregulation in proteins of the Wnt signaling pathway has also been implicated in multiple cancers, the most prominently described example being colorectal cancer (Arya et al, 2015; Symes et al, 2013; Zhan et al, 2017). Alterations in Wnt signaling have also been observed in cutaneous squamous cell carcinoma with which PeSCC is most analogous; these include upregulation of WNT5A and FZD6 and raised levels of β-catenin. Elevated levels of β-catenin have also been observed in lymphatic metastases of cutaneous squamous cell carcinoma (Sherwood AND Leigh, 2016).

Little was previously known about the role of Wnt signaling in PeSCC. Mentrikoski et al, (2014) found that cyclin D1 overexpression may contribute toward penile carcinogenesis. Papadopoulos et al (2007) reported overexpression of cyclin D1 in 61.9% of PeSCCs and a relationship between cyclin D1 expression and tumor differentiation. Arya et al (2015) remarked that all the Wnt signaling proteins tested (Wnt4, MMP7, cyclin D1, and c-MYC) were raised (by 1.6–3 folds) in PeSCC samples compared with those in normal controls. In addition, they reported significant differences between the colocalization of all 4 proteins tested in malignant tissues against controls (Arya et al, 2015). Our work agrees with these findings (statistically significant increases of 1.8–2.5 fold in the expression of all 4 studied proteins in PeSCC compared with those in normal skin) and multiple alterations in the colocalization of the studied protein.

In their report, Arya et al (2015) did not distinguish between PeSCC subtypes, combining HPV-driven and non–HPV-driven tumors in their analysis. This contrasts with the histological distinctions outlined in this study. Although our work has investigated the expression of the same set of Wnt signaling proteins as those reported by Arya et al (2015), it has focused specifically on HPV-independent PeSCC and its presumed precursor lesions, dPeIN and MGLSc. However, the changes in Wnt signaling observed in MGLSc and dPeIN and the progressive increase in the expression of cyclin D1 and c-MYC from MGLSc to dPeIN and ‘usual’ PeSCC provide intriguing hints about the pathway to HPV-independent penile carcinogenesis but do not presently provide any hard evidence in support of this.

In conclusion, our study highlights the differential expression and colocalization of Wnt signaling proteins across the spectrum of MGLSc, dPeIN, and PeSCC. Whereas MGLSc demonstrates localized upregulation of Wnt4 and minimal alterations in protein colocalization, PeSCC exhibits widespread dysregulation of Wnt signaling, characterized by increased protein expression and significant alterations in colocalization.

Although no definitive evidence was found for a direct role of Wnt signaling in the progression from MGLSc to PeSCC, the observed changes in protein expression and interaction patterns offer valuable insights into disease pathogenesis. Furthermore, the distinct expression profiles and colocalization patterns of Wnt-related proteins suggest their potential as diagnostic markers and therapeutic targets.

Future research should aim to validate these findings in larger cohorts and explore the therapeutic implications of modulating Wnt signaling in HPV-independent penile neoplasia. By advancing our understanding of the molecular mechanisms underlying these conditions, we can pave the way for more targeted and effective clinical interventions. In particular, future studies should investigate additional Wnt/β-catenin–target genes and compare expression patterns between PeIN and PeSCC cases with and without underlying lichen sclerosus. Finally, further studies should also compare Wnt signaling protein expression between HPV-associated and HPV-independent PeSCC to determine whether Wnt pathway dysregulation differs between these carcinogenic pathways.

## Materials and Methods

### Ethical approval and sample collection

Ethical approval (REC ref 20/SC/0037) was obtained from the joint research office at University College London Hospitals and University College London through the NHS Health Research Authority, South Central – Berkshire B Research Ethics Committee.

### Sample collection

Archival, formalin-fixed and paraffin embedded tissues were used from adult patients with a histopathological diagnosis of MGLSc, dPeIN and undifferentiated PeIN, and PeSCC made by 2 independent histopathologists, as described in detail elsewhere (Kravvas et al, 2024). The samples were collected from participants who had undergone either circumcision or excision of pathological lesions at University College London Hospitals as part of their routine management. All subjects gave written consent to access of their medical records and tissue samples. The demographics of each group are summarized in Table 4 and described in detail in Table 5.

**Table 4:** Patient Demographics according to Condition

Condition	Median Age of Diagnosis, y	Interquartile Range
MGLSc	53	43–65
dPeIN	69	51–73
PeSCC	66	51–73

Abbreviations: dPeIN, differentiated penile intraepithelial neoplasia; MGLSc, male genital lichen sclerosus; PeSCC, penile squamous cell carcinoma; uPeIN, undifferentiated penile intraepithelial neoplasia.

Tissue samples were obtained from patients with MGLSc (N = 48, n = 48), dPeIN (N = 21, n = 25), uPeIN (N = 22, n = 28), and ‘usual’ PeSCC (N = 23, n = 23). The mean age of diagnosis and associated interquartile ranges are provided for each condition (N = number of patients; n = number of tissue cores. Note: all PeSCC are of the ‘usual’ subtype).

**Table 5:** Detailed Patient Demographics

Patient Identifier	Diagnosis	PeSCC Subtype	Age at Diagnosis, y
A001	MGLSc	n/a	40
A002	MGLSc	n/a	63
A003	MGLSc	n/a	59
A004	MGLSc	n/a	37
A005	MGLSc	n/a	48
A006	MGLSc	n/a	56
A007	MGLSc	n/a	71
A010	MGLSc	n/a	61
A011	MGLSc	n/a	88
A012	MGLSc	n/a	29
A014	MGLSc	n/a	25
A015	MGLSc	n/a	34
A017	MGLSc	n/a	63
A018	MGLSc	n/a	61
A020	MGLSc	n/a	41
A021	MGLSc	n/a	54
A022	MGLSc	n/a	40
A023	MGLSc	n/a	56
A024	MGLSc	n/a	51
A026	MGLSc	n/a	53
A030	MGLSc	n/a	31
A031	MGLSc	n/a	62
A032	MGLSc	n/a	56
A033	MGLSc	n/a	68
A039	MGLSc	n/a	44
A040	MGLSc	n/a	47
A041	MGLSc	n/a	49
A042	MGLSc	n/a	82
A043	MGLSc	n/a	80
A045	MGLSc	n/a	42
A046	MGLSc	n/a	55
A049	MGLSc	n/a	33
A051	MGLSc	n/a	39
A052	MGLSc	n/a	24
A053	MGLSc	n/a	50
A055	MGLSc	n/a	51
A058	MGLSc	n/a	78
A060	MGLSc	n/a	47
A062	MGLSc	n/a	79
A064	MGLSc	n/a	73
A071	MGLSc	n/a	67
A075	MGLSc	n/a	48
A076	MGLSc	n/a	71
A077	MGLSc	n/a	55
A078	MGLSc	n/a	71
A079	MGLSc	n/a	51
A080	MGLSc	n/a	53
A081	MGLSc	n/a	79
B002	dPeIN	n/a	47
B003	dPeIN	n/a	48
B004	dPeIN	n/a	82
B005	dPeIN	n/a	39
B006	dPeIN	n/a	73
B007	dPeIN	n/a	71
B008	dPeIN	n/a	67
B011	dPeIN	n/a	69
B012	dPeIN	n/a	72
B016	dPeIN	n/a	71
B017	dPeIN	n/a	55
B018	dPeIN	n/a	71
B019	dPeIN	n/a	51
B023	dPeIN	n/a	51
B024	dPeIN	n/a	51
B025	dPeIN	n/a	79
B027	dPeIN	n/a	66
B028	dPeIN	n/a	77
B029	dPeIN	n/a	81
B031	dPeIN	n/a	67
B032	dPeIN	n/a	48
D007	PeSCC	Usual	32
D008	PeSCC	Usual	78
D015	PeSCC	Usual	59
D016	PeSCC	Usual	70
D020	PeSCC	Usual	48
D030	PeSCC	Usual	83
D034	PeSCC	Usual	67
D035	PeSCC	Usual	51
D044	PeSCC	Usual	73
D049	PeSCC	Usual	82
D051	PeSCC	Usual	67
D060	PeSCC	Usual	51
D064	PeSCC	Usual	64
D069	PeSCC	Usual	51
D071	PeSCC	Usual	54
D074	PeSCC	Usual	77
D077	PeSCC	Usual	69
D079	PeSCC	Usual	61
D080	PeSCC	Usual	62
D081	PeSCC	Usual	72
D086	PeSCC	Usual	48
D087	PeSCC	Usual	83

Abbreviations: dPeIN, differentiated penile intraepithelial neoplasia; MGLSc, male genital lichen sclerosus; n/a, not available; PeSCC, penile squamous cell carcinoma.

### Sample size calculation

A preliminary sample size calculation was performed for binary outcomes (ie, positive or negative) using a significance level (α = 5%) and a power (1−β = 90%). The percentage success in the control group was set at 5%, whereas the percentage success in the experimental group was set at 75%. This calculation determined that a sample size of at least 6 per group was required (Kravvas et al, 2024).

### Tissue array construction

A manually operated tissue arrayer (MTA1, Beecher Instruments) was used to construct tissue arrays using well-described techniques (Nariculam et al, 2009; Symes et al, 2013; Wang et al, 2010). Individual tissue cores of 1 mm in diameter and 6 mm in depth were extracted from the marked regions of MGLSc, dPeIN, PeSCC, and MGLSc/dPeIN-adjacent (normal) penile tissues (Kravvas et al, 2024). One or 2 cores were extracted from each tissue block. These “donor” cores were then deposited into recipient wax blocks and were sectioned at 6–8 μm thickness on glass slides using an automatic microtome (HM 355S, Thermo Fisher Scientific). Additional sections were stained with H&E for histological analysis by expert pathologists.

### Tissue array staining

The expression of 4 proteins of the Wnt pathway—Wnt4, MMP7, cyclin D1, and c-MYC—were assessed using multilabel fluorescence technique described in detail elsewhere (Arya et al, 2015; Xie et al, 2025). The following primary antibodies were used: anti-Wnt4 (1:200, polyclonal rabbit IgG, ab150596, Abcam), anti-MMP7 (1:40, monoclonal rabbit IgG, ab271977, Abcam), anti–cyclin D1 (1:40, polyclonal rabbit IgG, sc-718, CiteAb), anti–c-Myc (1:1000, monoclonal mouse IgG, NCL-cmyc, Novocastra).

Each antibody was optimized for pH and concentration dependence, antigen retrieval, and temperature parameters. The antibodies were labeled with Opal 480 (Wnt4), Opal 520 (MMP7), Opal 570 (cyclin D1), and Opal 650 (c-Myc), according to protocols described previously (Arya et al, 2015; Xie et al, 2025). All staining was carried out on a Leica RX research staining robot (Leica Biosystems) using published techniques (Arya et al, 2015; Giuliano et al, 2016; Leach, 1946; Symes et al, 2013; Wang et al, 2010).

### Tissue core imaging

**Figure 11:**
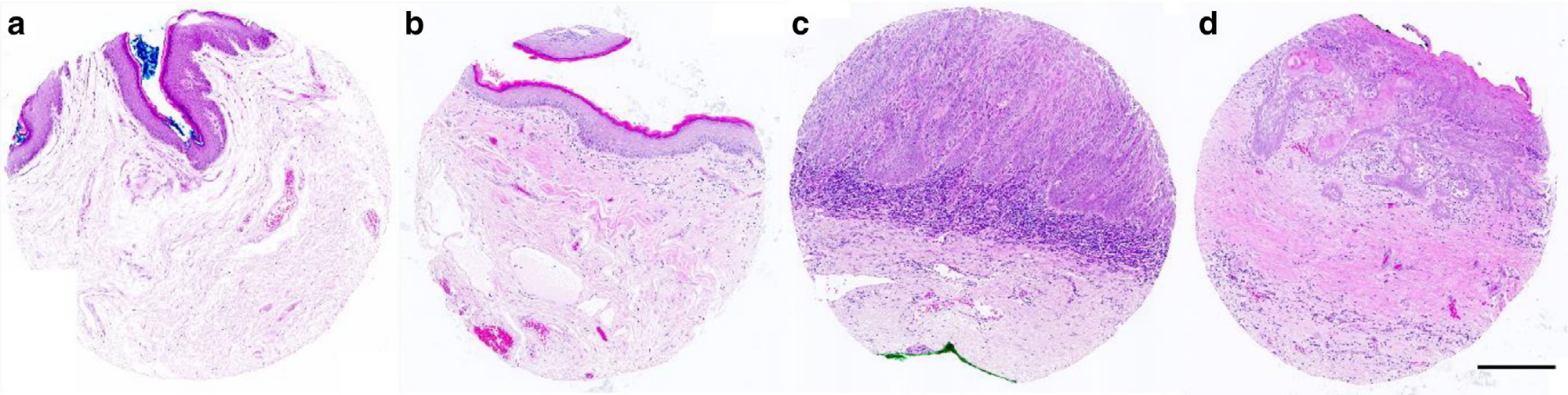
**H&E-stained tissue cores of normal penile skin, MGLSc, dPeIN, and PeSCC.** Representative images of H&E-stained tissue with disease types used in this study are shown. (**a**) Normal penile skin, (**b**) MGLSc, (**c**) dPeIN, and (**d**) PeSCC. Consistent settings were applied across all images to allow for comparative analysis. Each tissue core has a diameter of 1 mm. The images have been adapted from a previous figure utilizing the same tissue array for a different study. Bar = 600 μm. dPeIN, differentiated penile intraepithelial neoplasia; MGLSc, male genital lichen sclerosus; PeSCC, penile squamous cell carcinoma.

The H&E-stained slides were scanned under ×20 magnification with brightfield illumination using an AxioScan Z1 (Zeiss) slide scanner. Each individual core was then re-examined by a histopathologist to ensure the adequacy of each sample (Figure 11).

Typical histological signs of MGLSc include epidermal atrophy, edema and hyalinization of the superficial dermis, telangiectasia, and lichenoid chronic inflammation (Kravvas et al, 2018). Histologically, PeIN is characterized by dysplastic changes with an intact basement membrane. More specifically, dPeIN is characterized by dyskeratosis, acanthosis, and elongated rete ridges with marked atypia of the basal keratinocytes. Although the superficial maturation of the epithelium is conserved without atypia or koilocytes, there are atypical basal and parabasal keratinocytes containing abundant cytoplasm and hyperchromatic irregular nuclei with some mitotic figures. Occasionally, squamous pearls and dyskeratotic cells can also be seen at the tip of the rete ridges (Calonje et al, 2011; Kravvas et al, 2022bb). PeSCC histologically shows nests of atypical keratinocytes that arise in the epidermis and invade into the dermis, which displays areas of variable, aberrant, and ectopic keratinization and dyskeratosis (Calonje et al, 2011; Patterson, 2021). The usual type of PeSCC usually shows abundant keratinization with moderate differentiation. Foreign body–type giant cell reaction may be seen in response to keratinized tumors (Moch et al, 2016; Ro et al, 2020) (Figure 11).

The Zeiss AxioScan Z1 slide scanner with ×20 magnification was used to obtain whole-core images for protein expression analysis of the multiantibody fluorescence-labeled slides, as described elsewhere (Arya et al, 2015; Xie et al, 2025). Light intensity and camera exposure times were optimized for each fluorophore and kept uniform for all subsequent imaging (Table 6).

**Table 6:** A List of the Targeted Proteins and Associated Fluorophores Used in Multilabeled Slides

Protein	Wnt4	MMP7	Cyclin D1	c-MYC
Fluorophore	Opal 480	Opal 520	Opal 570	Opal 650
Excitation/Emission wavelength (nm) in AxioScan	440/498	495/520	553/568	587/617
Colour rendered in Zeiss AxioScan	Blue	Yellow	Red	Pink
Excitation/Emission wavelength (nm) in confocal	405/429	496/526	561/595	633/671
Colour rendered in Leica SP8	Blue	Yellow	Red	Pink

Abbreviation: MMP7, matrix metalloproteinase 7.

A list of the fluorophores used for each protein along with their rendered colur and excitation/emission values in both AxioScan and confocal microscopes is provided. Pseudo colors were used to render the images and help distinguish between adjacent wavelengths. The excitation/emission wavelength (nm) settings were adjusted between the AxioScan Z1 (Zeiss) and SP8 confocal (Leica) microscopes to yield the best quality images possible.

Smaller fields of the multilabeled tissue samples were imaged using a Leica SP8 confocal microscope (Leica biosystems) for protein colocalization analysis. Laser power, gain, and offset controls were optimized at the start of the experiment and kept uniform for all imaging. All tissue samples were imaged with ×64 optical zoom at a 1024 × 1024 pixel format (×63 1.4-numerical aperture oil objective lens). Z-slice sectioning was performed at 0.17 μm (at 600 Hz), yielding approximately 21–23 Z-sections. The small surface area of high-power images captured by the confocal microscope was visually divided into dermis and epidermis by a qualified histopathologist. This enabled the separate analysis of protein colocalization within the epidermis and dermis.

### Image analysis

**Figure 12:**
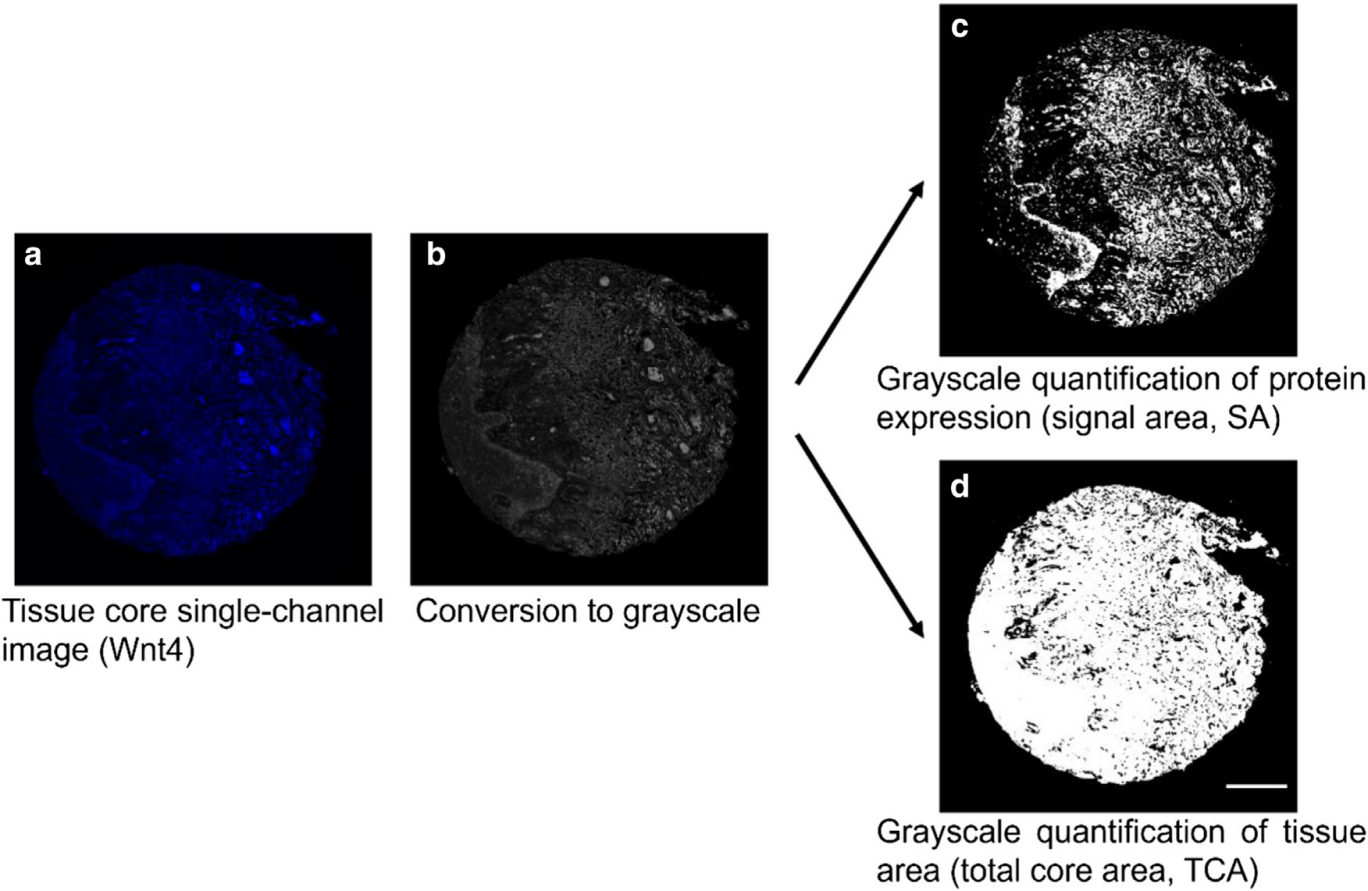
**Thresholding protocol for protein expression analysis by measuring fluorescence intensity.** (**a**) Photograph of a single tissue core stained for Wnt4 (blue, Opal 480 labeled). (**b**) The same image was converted to grayscale using Fiji software. (**c**) Thresholding was undertaken to quantify the SA. (**d**) Separate thresholding was undertaken to define the TCA. Protein expression was calculated by dividing the SA of each core by the TCA. Figure was adapted from Wang et al (2010). Bar = 600 μm. SA, signal area; TCA, total core area.

To quantify the expression levels of the proteins, we performed an unbiased, quantitative, and semiautomated image analysis using an adapted ImageJ plugin (Arya et al, 2015; Symes et al, 2013). The images of each tissue core were separated into their 4 individual channels—blue (Opal 480, Wnt4), yellow (Opal-520, MMP7), red (Opal-570, cyclin D1), and pink (Opal 650, c-MYC)—and channels and were exported from Zen 3.7 (Zeiss) software. The files were subsequently opened in ImageJ software and converted to 8-bit grayscale; a threshold and segmentation routine was then applied (Arya et al, 2015; Wang et al, 2010). Lower and upper threshold parameters were set to include the median to maximum gray values of a range of samples across the sample cohort. The gray values were then used to perform semiautomated analysis of the whole tissue array. Using this approach, we calculated the tissue area in each core and the area of protein expression in each core. The total amount of tissue in each core was divided by the area of protein expression to obtain a standardized signal of protein intensity for each label and in each tissue sample (Symes et al, 2013) (Figure 12).

For colocalization measurements, the confocal image files were deconvolved using Huygens Professional software (Scientific Volume Imaging). The deconvolved files were also analyzed by the Huygens professional software colocalization module to calculate Global Intersection Coefficients for each of the 6 protein pairs that arise by pairing the 4 fluorescent channels. The Global Intersection Coefficient is a commonly used measure of the proximity of 2 fluorophore-labeled entities and ranges from 0 (no overlap) to 1 (compete overlap) (Giuliano et al, 2016).

### Statistical analysis and data presentation

Statistical analysis was performed using MedCalc (MedCalc) software. Boxplots were made using Prism (GraphPad) software.

Data in the text are presented as median values with 25 and 75% interquartile ranges annotated within square brackets. In figures, the boxplots present the same ranges and, additionally, present minimum and maximum values. Differences between datasets were tested by ANOVA with posthoc, 2-tailed, unpaired, nonparametric Mann–Whitney *U* tests. Statistical correction was applied on all *P*-values using the Holm–Bonferroni method. The null hypothesis was rejected if the *P* < .05.

## Ethics Statement

This study was performed in accordance with the Declaration of Helsinki. Collection of human tissue samples for this study was approved as part of the study protocol. This human study was approved by University College London Hospitals/University College London joint research office (approval 20/SC/0037). All adult participants provided written informed consent to participate in this study.

## Data Availability Statement

Data are available on request (georgios.kravvas@nhs.net).

## ORCIDs

Georgios Kravvas: http://orcid.org/0000-0002-1924-0149

Boyu Xie: http://orcid.org/0000-0002-3966-3261

Michael Millar: http://orcid.org/0000-0003-4264-3568

Alex Freeman: http://orcid.org/0000-0001-5031-3791

Aiman Haider: http://orcid.org/0000-0002-7005-4245

Hussain M. Alnajjar: http://orcid.org/0000-0001-6364-0310

Asif Muneer: http://orcid.org/0000-0003-2958-1614

Aamir Ahmed: http://orcid.org/0000-0001-7405-5336

Christopher Barry Bunker: http://orcid.org/0000-0002-6693-748

## Conflict of Interest

The authors state no conflict of interest.
